# Economic burden of depressive disorders and HIV for people living with HIV in Uganda

**DOI:** 10.1093/heapol/czag054

**Published:** 2026-04-17

**Authors:** Patrick V Katana, Ian Ross, Barbra Elsa Kiconco, Patrick Tenywa, Melissa Neuman, Wilber Ssembajjwe, Isaac Sekitoleko, Kenneth Roger Katumba, Eugene Kinyanda, Yoko V Laurence, Giulia Greco

**Affiliations:** Department of Disease Control, London School of Hygiene and Tropical Medicine, Keppel Street, London WC1E 7HT, United Kingdom; Health Economics Research Unit, KEMRI Wellcome Trust Research Programme, Second Floor, 197 Lenana Place, PO BOX 43640-00100, Nairobi, Kenya; Department of Health Services Research and Policy, London School of Hygiene and Tropical Medicine, 15–17 Tavistock Place, London WC1H 9SH, United Kingdom; MRC/UVRI & LSHTM Uganda Research Unit, PO Box 49, Plot 51-59 Nakiwogo Road, Entebbe, Uganda; MRC/UVRI & LSHTM Uganda Research Unit, PO Box 49, Plot 51-59 Nakiwogo Road, Entebbe, Uganda; Department of Infectious Disease Epidemiology and International Health, London School of Hygiene and Tropical Medicine, Keppel Street, London WC1E 7HT, United Kingdom; MRC/UVRI & LSHTM Uganda Research Unit, PO Box 49, Plot 51-59 Nakiwogo Road, Entebbe, Uganda; MRC/UVRI & LSHTM Uganda Research Unit, PO Box 49, Plot 51-59 Nakiwogo Road, Entebbe, Uganda; MRC/UVRI & LSHTM Uganda Research Unit, PO Box 49, Plot 51-59 Nakiwogo Road, Entebbe, Uganda; Department of Global Health and Development, London School of Hygiene and Tropical Medicine, 15-17 Tavistock Place, London WC1H 9SH, United Kingdom; MRC/UVRI & LSHTM Uganda Research Unit, PO Box 49, Plot 51-59 Nakiwogo Road, Entebbe, Uganda; Department of Global Health and Development, London School of Hygiene and Tropical Medicine, 15-17 Tavistock Place, London WC1H 9SH, United Kingdom; Health Economics & Health Policy, Global Epidemiology and Modelling Bristol, University of Bristol, 39 Whatley Road, Bristol BS8 2PS, United Kingdom; Department of Global Health and Development, London School of Hygiene and Tropical Medicine, 15-17 Tavistock Place, London WC1H 9SH, United Kingdom

**Keywords:** HIV/AIDS, depression, economic burden, PHQ-9

## Abstract

Between 8%–39% of people living with HIV (PLWH) in sub-Saharan Africa have a depressive disorder (DD). Despite considerable gains in the treatment of PLWH, DD is increasingly recognised as a threat to successful treatment and prevention. PLWH incur higher health-related costs than the general population due to chronic care management needs. We aimed to estimate the combined economic burden of DD and HIV amongst PLWH and explore their mechanisms of coping with high-of-pocket health expenditure. This was a cost of illness study nested in a cluster-randomized trial that assessed the effectiveness of integrating treatment of DD into routine HIV care in Uganda (HIV+D trial). The study used cross-sectional data collected from 1115 PLWH across both trial arms at baseline, using the 9-item Patient Health Questionnaire (PHQ-9) to measure DD and a structured cost questionnaire. The mean monthly economic cost of HIV and DD amongst *n* = 486 participants reporting at least one non-zero cost item was United States Dollars (USD) 11.72 (2022 prices), while the mean across the whole sample (including zeroes) was USD 5.05. Mean monthly out-of-pocket expenditure amongst participants reporting at least one non-zero item was USD 7.22, which is 4% of average monthly household income. It was USD 3.11 in the sample as a whole. Moderate DD symptoms (PHQ-9 between 15–19) and severe symptoms (PHQ-9 ≥ 20) were reported by 30% and 5% of respondents respectively, with the remainder experiencing mild symptoms. Social protection mechanisms combined with the integration of the management of DD into routine HIV care could help alleviate this burden.

Key messages

- Despite the implementation of strategies to prevent HIV, depressive disorders remain a significant threat since they can impact treatment adherence.
- Increased life expectancy for people living with HIV due to improvements in treatment and care comes with the responsibility of lifelong caring costs.
- Although antiretrovirals are fully subsidised in Uganda, the economic burden arising from care and management of HIV and depression remains a public health concern.

## Introduction

Globally, ∼38 million people are living with HIV (PLWH) and of these, between 8%–39% have depressive disorders (DD) (Myer et al. 2008, Kinyanda et al. 2011, 2017, 2021). In Uganda, 31% of the estimated 1.4 million PLWH suffer from depression according to a meta-analysis (Wagner et al. 2017). Depression is a transient mood state experienced by individuals at some times in their life, and major DD affects mental health functioning. DD remains a significant threat to the fight against HIV, despite the tremendous progress made to improve access to HIV care in Uganda and the region. This is because DD may impact on HIV treatment adherence and risky sexual behaviour. Several studies have shown PLWH suffer from stress and incur higher health-related costs compared to the general population due to care management demands throughout their lifespan (Abas et al. 2014, Uthman et al. 2014).

The improved survival of PLWH in recent decades due to improvements in treatment and care represents huge progress, but comes with lifelong caring costs for their family and the state (Cohen et al. 2020, Gandhi et al. 2023). This requires PLWH and family members to bear costs that they are often unable to bear on their own. Such costs comprise direct medical (e.g. consultations, drugs), direct non-medical (e.g. transport and food) and indirect (e.g. reduced PLWH and caregiver productivity) costs. Antiretroviral therapy (ART) drugs for HIV/AIDS are free of charge for patients at public health centres in Uganda, and many parts of the world, so direct medical costs of HIV should in theory be zero. However, PLWH can still incur costs for comorbid conditions (Pinto et al. 2013). In particular, DD associated with HIV can increase overall costs of care through increased visits to health facilities when compared with PLWH without DD (Kinyanda et al. 2017).

Many PLWH still incur medical costs to treat opportunistic infections in addition to non-medical costs (Pinto et al. 2013). These costs may be catastrophic to people in fragile economic situations in the context of low insurance coverage (Barennes et al. 2015). To meet the costs of illness, PLWH and their caregivers may adopt coping strategies such as selling assets, or borrowing from relatives or a lending institution (Russell 1996, Sauerborn et al. 1996, McIntyre et al. 2006). Such strategies can help to cope with the immediate economic burden; however, they can also be potentially ‘risky’ for their future financial wellbeing. The selling of livelihood assets, for instance, reduces the caregiver’s or family’s ability to generate future income (Flores et al. 2008).

Several studies have focused on the economic burden of HIV on people’s wellbeing, quality of life, and mental health (Maheswaran et al. 2018, Jakubowski et al. 2022). However, no studies have evaluated the association between costs and severity of DDs for PLWH or evaluated indirect costs beyond PLWH, namely caregivers. It is against this background that we undertook a cost of illness assessment within a cluster-randomized trial of integrated management of depression into routine HIV care (HIV+D). Using the cost data collected at baseline, this study sought to estimate, amongst Ugandan PLWH who also had DD, the combined economic burden of DD and HIV.

## Methods

### Study setting

Uganda is a low-income country with a population of 45 million people and a 2024 gross national income per capita of United States Dollars (USD) 1020 (World Bank 2025). It is estimated that 30% of this population lives below the poverty level of USD 1.77 per person per day (UBOS 2021). A strong association between depression and poverty has been shown, and PLWH are at a much higher risk (Cleary et al. 2020, Rutakumwa et al. 2023). In addition, 9.8% of Uganda’s gross domestic product is spent on healthcare but only 1% of total health expenditure is allocated to mental health (Molodynski et al. 2017). Despite access to free HIV care at point of delivery, PLWH continue to incur costs related to HIV and resulting comorbidities (Kakaire et al. 2016).

The HIV+D study was a cluster-randomized trial (Kinyanda et al. 2021) that examined effectiveness and cost-effectiveness of integrating the management of depression into routine HIV care (ISRCTN registration ISRCTN86760765). It was conducted in 40 public health facilities that provide HIV care in semi-urban and rural settings of central and southwestern Uganda with a high prevalence of HIV (Ssebunnya et al. 2021). PLWH who were ≥18 years old and had been on ART for at least 6 months were eligible. Between 10 and 30 eligible participants were recruited by random sampling from each of the 40 facilities in the districts of Kalungu, Masaka, and Wakiso, with a total of 1200 PLWH enrolling (Kinyanda et al. 2026).

### DDs

The 9-item Patient Health Questionnaire (PHQ-9) (Kaggwa et al. 2022) was administered to screen for eligibility in the trial at baseline. The PHQ-9 provides an indicator of depressive symptom severity, with items scored on a 4-point Likert scale from 0 (not at all) to 3 (nearly every day). Total scores are a sum of item scores, ranging from 0 to 27 (worst possible). As a measure of severity, PHQ-9 scores are interpreted as 0–4 (minimal DD), 5–9 (minor), 10–14 (mild), 15–19 (moderate), and ≥20 (severe). The trial used a cut-off ≥10 for eligibility, which maximized sensitivity and specificity in another study conducted in Uganda (Akena et al. 2013). To assess the association between cost and PHQ-9 score, we divide the PHQ-9 score into two groups: mild (10–14) and moderate/severe (≥15).

### Study design

This was a cross-sectional study nested within the HIV+D trial. Cost data for the trial was collected from PLWH at baseline, 3 months, and 12 months through face-to-face interviews at public health facilities, but the present study is retrospective using baseline data. Costs were estimated bottom-up from the societal perspective. The time horizon was the previous month. For this study, we used only the baseline data from the study in a sample of 1115 participants across both trial arms. At the time of questionnaire design, likely cost centres and inputs were enumerated within the team, and a category for ‘other’ was used in case costs were in-scope but did not fit into a category.

### Data collection

Adults aged ≥18 years with HIV and mild depression or above were recruited from 40 randomly selected primary HIV care centres in three districts of Uganda. Participants were required to be on ART for at least 6 months, medically stable, conversant in English or Luganda, willing to be visited at home, expected to remain in the study area for 12 months, and able to provide informed consent. Exclusion criteria included impairments that hindered engagement with research procedures, current treatment for depression or psychiatric conditions, and alcohol use problems. DD was measured using the PHQ-2 for initial screening, with scores ≥3 prompting further eligibility assessment using the PHQ-9 to confirm depression before recruitment. We only sampled outpatient clients (HIV clinic attendees) who had come to ART clinics in the health facilities for their routine HIV care visits. Inpatient clients were not sampled. Out of 8441 HIV clinic attendees approached to take part in the study, 1115 were enrolled after meeting the eligibility criteria for inclusion.

We developed a questionnaire that was used to retrospectively collect costs incurred by PLWH at the baseline of the HIV+D study from 4 May 2021 to 17 December 2021. The cost questionnaire was translated from English to Luganda (the predominantly used local language) and then back translated to ensure accuracy and coherence. This was done by the HIV+D Health Economics team in consultation with social scientists who were not part of the trial and a psychologist who was a member of the HIV+D study team. All were experienced with translation of research questionnaires. This baseline cost questionnaire contained questions on direct and indirect costs, visit type (outpatient and inpatient), household income, household expenditure, and sociodemographic information. Recall was limited to the previous month so as to minimize recall bias. The questionnaire also covered coping strategies (which would be ‘not applicable’ if there were no costs at all), such as borrowing, use of savings, or selling household assets. In cases of borrowing, the source of any borrowed resources was asked (e.g. from friends, a bank, or other sources) as well as any interest charged on loans. A paper questionnaire was administered through face-to-face interviews lasting 30–40 min. Completed questionnaires were reviewed by the research team for missing information, then data was entered into the study database in OpenClinica for cleaning and analysis.

### Data analysis

We applied a cost of illness approach and included direct and indirect costs. Direct costs were measured by combining out-of-pocket (OOP) medical and non-medical expenditure for inpatient and outpatient visits. These included administrative costs e.g. consultation fees for clinicians, daily hospital charges, user fees, HIV and DD diagnostic test fees, the cost of HIV and DD drugs, supplementary foods, and return travel to the health care facility for diagnostic and treatment visits. In addition to regular visits to the HIV clinics, some PLWH also visited the health care facility for separate depression treatment. In our study, the costs attributable to both HIV and depression visits were combined. For example, the healthcare utilization question asked: ‘In the past month, how many times have you accessed care for symptoms related to depression or anxiety or for HIV care at the following places?’ (then listing different kinds of healthcare facilities). We did not think it feasible to measure them separately in a way that was meaningful without confusing participants. Furthermore, the cost of illness analysis is an input to a cost-effectiveness analysis, so the rationale was that the intervention could also affect HIV-related costs as well as DD-related costs, and capturing incremental costs overall is the priority. Relatively few people were expected to report direct medical costs as compared to travel costs and productivity loss because HIV care is free in Uganda.

Indirect costs were estimated by capturing the loss of productive working time and calculating wages using the human capital approach. Productivity losses by PLWH and caregivers or accompanying persons (as reported by participants) was estimated by multiplying the number of days lost due to DD (which included time for seeking or receiving care or periods of inability to work) by a value for time. We assumed an average adult will work for 8 hr in a day, so a day’s wage lost is equivalent to 480 min. To apply a common denominator, all income (in cash or in kind) was converted to a daily rate by using the assumptions of 5 working days in a week on average, 20 in a month, and 260 in a year. If the participant earned a wage, their time was valued using the average of their reported income over the past month from the primary job divided by 20 days. If they had secondary job(s), then we summed the reported income across all jobs to obtain the total daily wage of the person over the past month. Average productivity costs were calculated by multiplying that daily wage by the number of days lost as a result of seeking and receiving HIV and depression care.

The average cost of HIV and DD care was computed as the sum of direct and indirect costs over the duration of 1 month. Our primary results are the mean cost [with standard deviation (SD)] and median [with interquartile range (IQR)]. We report means/medians where the denominator is those incurring costs (excluding zero costs) and also where the denominator is the sample as a whole (*n* = 1115). The second of these is a sensitivity analysis which effectively interprets missing values as zero costs, which we believe is justified from the way the questionnaire was formatted and data entered. Costs were collected in Ugandan shillings (UGX) and converted into USD using the average exchange rate across the year from XE.com 1 USD = 3687 UGX for 2022.

We used descriptive statistics to summarise all socio-demographic, visit type, coping strategies, and depressive symptoms variables. Means and medians of continuous data and proportions for categorical data were summarised. We used principal component analysis of asset ownership variables to generate a wealth index and quintiles for the whole household sample (Vyas and Kumaranayake 2006). Since the cost data were highly skewed, we used a generalized linear regression model with gamma family (Veazie et al. 2023) and log link to assess the association between cost and PHQ-9 score, a measure of depression severity, and accounted for clustering by facility. Alternative specifications were considered, along with the Bayesian information criterion and distribution of residuals to inform model selection.

## Results

A total of 1115 respondents completed the structured cost questionnaire. The majority were female (77%), which approximately aligns with the distribution of HIV in Uganda which is higher among women (7.2%) than men (4.3%) (Table 1). The mean age of participants was 39 years (SD = 12.0) and 39% had no formal education. Approximately half of the participants (48%) were married or living with someone and the majority were Christians (86%). The mean PHQ-9 score was 13.7 (SD = 3.1). About a quarter (24%) engaged in an income-generating activity in the past 1 month. Coping strategies for the economic costs of HIV and depression were reported by 20% of participants.

**Table 1:** Baseline socio-demographic and clinical characteristics of people with HIV and depression in Uganda.

	Gender		PHQ-9 score		Total *n* (%)
	Female *n* (%)	Male *n* (%)	Mild (10–14) *n* (%)	Moderate or severe (≥15) *n* (%)	
**Characteristic (*n* = 1115)**	859 (77.0)	256 (23.0)	728 (65.3)	387 (34.7)	1115 (100)
**Age (years)**					
18–25	120 (14.2)	20 (7.9)	88 (12.3)	52 (13.6)	140 (13)
26–35	311 (36.8)	53 (20.9)	237 (33.1)	127 (33.2)	364 (33)
36–45	230 (27.2)	85 (33.6)	200 (27.9)	115 (30.1)	315 (29)
46–55	121 (14.3)	66 (26.1)	125 (17.4)	62 (16.2)	187 (17)
56+	64 (7.6)	29 (11.5)	67 (9.3)	26 (6.8)	93 (8)
**Educational attainment**					
No formal education	349 (40.6)	89 (34.8)	283 (38.9)	155 (40.1)	438 (39)
Primary	371 (43.2)	109 (42.6)	324 (44.5)	156 (40.3)	480 (43)
Secondary	118 (13.7)	49 (19.1)	104 (14.3)	63 (16.3)	167 (15)
Tertiary education	21 (2.4)	9 (3.5)	17 (2.3)	13 (3.4)	30 (3)
**Employment status**					
Engaged in an income-generating activity in past month	124 (14.6)	29 (11.5)	95 (13.2)	58 (15.1)	153 (14)
Not engaged in an income-generating activity	724 (85.4)	224 (88.5)	623 (86.8)	325 (84.9)	948 (86)
**Marital status**					
Single	85 (9.9)	39 (15.2)	72 (9.9)	52 (13.4)	124 (11)
Married	391 (45.5)	145 (56.6)	346 (47.5)	190 (49.1)	536 (48)
Widowed	122 (14.2)	12 (4.7)	101 (13.9)	33 (8.5)	134 (12)
Separated or divorced	261 (30.4)	60 (23.4)	209 (28.7)	112 (28.9)	321 (29)
**Religion**					
Catholic	392 (45.6)	141 (55.1)	352 (48.4)	181 (46.8)	533 (48)
Protestant	328 (38.2)	328 (38.2)	328 (38.2)	153 (39.5)	226 (20)
Muslim	139 (16.2)	25 (9.8)	111 (15.2)	53 (13.7)	164 (15)
**PHQ-9 score (mean/SD)**					
Mean	13.57	14.15	11.86	17.18	13.70
(Median, SD)	(13, 3.06)	(14, 3.37)	(12, 1.49)	(16, 2.39)	(13, 3.14)
**Used coping strategies**					
Yes	176 (20.5)	40 (15.7)	136 (18.7)	80 (20.8)	216 (19)
No	682 (79.5)	214 (84.3)	591 (81.3)	305 (79.2)	896 (81)
**District**					
Kalungu	143 (16.6)	37 (14.5)	128 (17.6)	52 (13.4)	180 (16)
Masaka	187 (21.8)	75 (29.3)	172 (23.6)	90 (23.3)	262 (23)
Wakiso	529 (61.6)	144 (56.2)	428 (58.8)	245 (63.3)	673 (60)

### Economic costs

We report monthly average cost results in two ways, which are useful for different purposes. First, we report itemized means/medians for cost items amongst those incurring cost for that item, so zero costs are excluded from the calculation (Table 2). Second, we report itemized means/medians across the sample as a whole, where the denominator is *n* = 1115 (Table 3). About half the sample (44%) reported at least one non-zero cost item, and the mean economic cost of HIV and DD amongst these 486 individuals was USD 11.72 per month (SD 60.26), with median USD 2.17 per month (IQR 1.08–5.29). The mean economic cost of HIV and DD across the *n* = 1115 sample as a whole was USD 5.05 (SD 40.22) per month, with median USD 0.00 (IQR = 0–1.61). Relatively few people reported direct medical costs whereas many reported travel costs in particular, as well as productivity loss (Table 2). For 83% of the *n* = 486 reporting at least one non-zero cost item, travel cost was the only cost item. This contributed to direct cost contributing 92% of total cost on average for those *n* = 486.

**Table 2:** Itemized mean and median monthly economic cost of HIV and DD amongst those incurring costs for that item, where the denominator per item is the number of people reporting non-zero cost for that item (USD and UGX).

	*n* Reporting non-zero cost per item	Mean monthly cost per person		Median USD [IQR]
		UGX (SD)	USD (SD)	
**Direct medical costs**				
Daily bed charges	28	102 929 (200 022)	27.27 (52.99)	10.60 [5.56–39.74]
Consultation fees	24	14 208 (11 209)	3.76 (2.97)	2.65 [1.32–5.30]
User fees	21	22 214 (24 323)	5.89 (6.45)	2.65 [1.86–7.95]
Laboratory fees	25	16 400 (11 662)	4.35(3.09)	5.30 [1.33–5.30]
Radiology fees	6	40 000 (30 332)	10.60 (8.04)	7.95 [5.30–10.60]
Other procedures	13	16 039 (18 054)	4.25 (4.79)	2.65 [1.33–5.30]
HIV drugs	12	40 667 (113 356)	10.78 (30.05)	1.46 [1.19–4.64]
Depression drugs	34	26 132 (39 094)	6.98 (10.45)	4.01 [1.87–6.68]
Other drugs	33	11 879 (11 238)	3.18 (3.00)	1.60 [1.34–4.01]
Supplements	22	27 818 (46 347)	7.44 (12.39)	2.67 [2.14–4.81]
Other medical costs	30	22 733 (31 321)	6.08 (8.37)	2.87 [1.87–8.02]
**Direct non-medical costs**				
Travel	455	8458 (10 691)	2.26 (2.86)	1.60 [1.07–2.67]
Food	37	17 446 (29 424)	4.66 (7.86)	1.34 [0.80–5.35]
Other non-medical costs	36	19 311 (36 427)	5.16 (9.74)	1.20 [0.29–5.35]
**Indirect costs**				
Productivity loss and wages	78	105 216 (472 308)	28.22 (126.69)	2.95 [0.52–9.39]

**Table 3:** Itemized mean and median monthly economic cost of HIV and DD in the whole sample, where the denominator per item is all 1115 participants (USD and UGX).

	Total *n*	Mean monthly cost per person		Median USD [IQR]
		UGX (SD)	USD (SD)	
**Direct medical costs**				
Daily bed charges	1115	2585 (36 061)	0.70 (9.51)	0 [0–0]
Consultation fees	1115	305 (2617)	0.08 (0.710)	0 [0–0]
User fees	1115	418 (4444)	0.11(1.205)	0 [0–0]
Laboratory fees	1115	368 (2972)	0.10 (0.81)	0 [0–0]
Radiology fees	1115	215 (3564)	0.06 (1.07)	0 [0–0]
Other procedures	1115	187 (2545)	0.05 (0.69)	0 [0–0]
HIV drugs	1115	438 (12 021)	0.12 (3.26)	0 [0–0]
Depression drugs	1115	796 (8092)	0.22 (2.20)	0 [0–0]
Other drugs	1115	352 (2772)	0.10 (0.75)	0 [0–0]
Supplements	1115	549 (7448)	0.15 (2.02)	0 [0–0]
Other medical costs	1115	612 (6251)	0.17 (1.70)	0 [0–0]
**Direct non-medical costs**				
Travel	1115	3452 (7992)	0.94 (2.17)	1.36 [0–1.36]
Food	1115	579 (6144)	0.16 (1.67)	0 [0–0]
Other non-medical costs	1115	624 (7304)	0.17 (1.98)	0 [0–0]
**Indirect costs**				
Productivity loss and wages	1115	7360 (127 043)	2.00 (34.46)	0 [0–0]

The mean OOP cost amongst people incurring at least one non-zero cost item was USD 7.22 per month, equating to ∼4% of the average monthly income of Ugandan households (USD 175). It was USD 3.11 in the sample as a whole. OOP cost is the same as direct cost in our case, since only costs amongst PLWH were included, and not those borne by the health system. Considering productivity loss, the average number of days lost due to HIV and depression amongst participants and caregivers was 6 days per month (amongst those losing >0 days), and the economic value of those losses was USD 28.22 per month on average (Table 2).

### Association with severity of DD

A total of 726 (65%), 330 (30%), and 57 (5%) of participants had mild, moderate, and severe depressive symptoms respectively. Tables 4 and 5 compare costs for those with moderate/severe depression (35%) to those with mild symptoms at the item level. Amongst the *n* = 486 incurring non-zero costs, regression results showed that total cost was not statistically significantly different between people with moderate/severe DD as compared to mild DD (*P* = 0.974).

**Table 4:** Itemized mean and median monthly economic cost (USD) of HIV and DD amongst those incurring costs for that item, sub-categorized by level of depressive symptoms (denominator is people reporting non-zero cost for that item).

	Mild depressive symptoms (PHQ score 10–14)			Moderate and severe depressive symptoms (PHQ score ≥15)		
	*n*	Mean costs (SD)	Median costs [IQR]	*n*	Mean costs [SD]	Median costs [IQR]
**Direct medical costs**						
Daily charges	20	18.19 (28.63)	10.69 (11.76)	8	50.82 (89.05)	21.38 (34.75)
Consultation fees	13	3.47 (2.09)	2.67 (4.01)	11	4.18 (3.89)	2.67 (4.01)
User fees	14	6.62 (7.70)	3.74 (8.02)	7	4.58 (3.05)	2.67 (5.88)
Laboratory fees	15	3.74 (2.47)	2.67 (4.01)	10	5.35 (3.83)	5.35 (5.35)
Radiology fees	4	12.70 (9.61)	9.36 (12.03)	2	6.68 (1.89)	6.68 (2.67)
Other procedures	9	5.00 (5.62)	2.67 (4.01)	4	2.67 (2.00)	2.41 (2.67)
HIV drugs	8	2.17 (2.02)	1.34 (2.27)	4	28.27 (52.45)	2.81 (54.39)
Depression drugs	22	6.88 (7.63)	4.68 (5.35)	12	7.17 (14.71)	3.34 (3.61)
Other drugs	21	2.88 (2.43)	1.60 (2.67)	12	3.70 (3.88)	2.14 (4.41)
Supplements	15	7.77 (13.45)	2.67 (3.21)	7	6.72 (10.68)	2.67 (3.47)
Other medical costs	20	5.21 (6.41)	2.67 (5.21)	10	7.80 (11.58)	3.88 (5.35)
**Direct non-medical costs**						
Travel	300	2.20 (2.10)	1.60 (1.60)	155	2.38 (3.94)	1.60 (1.60)
Food	29	5.42 (8.72)	2.41 (4.81)	8	1.90 (1.77)	1.20 (1.87)
Other non-medical costs	27	4.13 (8.01)	1.07 (5.08)	9	8.27 (13.84)	1.34 (4.81)
**Indirect costs**						
Productivity loss	47	16.73 (60.50)	2.01 (11.57)	31	35.80 (155.98)	3.35 (8.68)

**Table 5:** Itemized mean and median monthly economic cost (USD) of HIV and DD in the whole sample, sub-categorized by level of depressive symptoms (denominator is all participants in that symptom category).

	Mild depressive symptoms (PHQ score 10–14)			Moderate and severe depressive symptoms (PHQ score ≥15)		
	*n*	Mean costs. USD (SD)	Median costs [IQR]	*n*	Mean costs [IQR]	Median costs [IQR]
**Direct medical costs**						
Daily charges	726	0.51 (5.59)	0 (0)	387	1.07 (14.21)	0 (0)
Consultation fees	726	0.06 (0.54)	0 (0)	387	0.12 (0.95)	0 (0)
User fees	726	0.13 (1.40)	0 (0)	387	0.08 (0.73)	0 (0)
Laboratory fees	726	0.08 (0.64)	0 (0)	387	0.14 (1.05)	0 (0)
Radiology fees	726	0.07 (1.14)	0 (0)	387	0.03 (0.50)	0 (0)
Other procedures	726	0.06 (0.82)	0 (0)	387	0.03 (0.33)	0 (0)
HIV drugs	726	0.02 (0.31)	0 (0)	387	0.30 (5.52)	0 (0)
Depression drugs	726	0.21 (1.78)	0 (0)	387	0.23 (2.82)	0 (0)
Other drugs	726	0.08 (0.64)	0 (0)	387	0.12 (0.93)	0 (0)
Supplements	726	0.16 (2.20)	0 (0)	387	0.12 (1.63)	0 (0)
Other medical costs	726	0.15 (1.36)	0 (0)	387	0.21 (2.19)	0 (0)
**Direct non-medical costs**						
Travel	726	0.91 (1.75)	0 (0)	387	0.97 (2.79)	0 (0)
Food	726	0.04 (0.37)	0 (0)	387	0.22 (2.06)	0 (0)
Other non-medical costs	726	0.16 (1.73)	0 (0)	387	0.20 (2.39)	0 (0)
**Indirect costs**						
Productivity loss	726	2.34 (40.72)	0 (0)	387	1.36 (17.66)	0 (0)

### Coping strategies

Overall, 19% of study participants employed one or more coping strategy to cover the economic costs of seeking or receiving care for HIV and DD, the majority (73%) of whom borrowed from family and friends. The amount paid back was greater than the amount borrowed for most participants, and 89% were yet to fully repay the borrowed amount.

## Discussion

This study provides evidence of the combined economic burden of HIV and DD amongst PLWH who were participants in the HIV+D study. Overall, our results indicate an economic burden of USD 11.72 per person per month amongst people incurring any cost (44% of the sample) or USD 5.05 in the sample as a whole. The mean direct cost (i.e. the same as OOP in our study) amongst those incurring any costs was USD 7.22 per month, and USD 3.11 in the whole sample. The former estimate is lower than direct costs reported in another study on HIV/AIDS and mental health conducted in Nepal (Poudel et al. 2017). That study found an average direct cost of USD 20.4 (2011 prices), which would be around USD 40 in 2022 after adjusting for inflation. Our former estimate is also lower than that of a study on HIV/AIDS and mental health in Kenya (Katana et al. 2020), which evaluated costs for caregivers of adolescent PLWH. That study found an average direct cost of USD 16.72 (2018 prices) which would be around USD 20 in 2022. The discrepancy could be down to a number of factors, e.g. differences in the nature of the health system and which HIV-related costs are covered by the state, and that the Kenyan study focused on costs for caregivers only. It could also be related to zeroes and missing values in our data, as discussed further below.

Bed charges were an important contributor to cost both amongst people incurring those charges (Table 2) and across the sample (Table 3). Although an individual living with HIV may suspect that they have a DD, they might not visit the hospital or clinic until they are very ill because of possible discrimination from their family, neighbours, or society (Bwanika et al. 2022). Moreover, health insurance coverage in Uganda is low, meaning many individuals pay OOP for much of their treatment, further incentivizing delaying care until symptoms are severe. These delays in care-seeking may exacerbate their healthcare needs and the likely need for costly inpatient care. We hypothesise that many of the hospitalizations could have resulted from opportunistic infections, hence the need for ART and adherence-enhancing interventions to reduce vulnerability (Holmes et al. 2006, Pinto et al. 2013). Productivity loss is likely to be correlated with hospitalization, and the value of lost productivity was sizeable in relation to other items, whether amongst those incurring costs (Table 2) or in the whole sample (Table 3).

People living with HIV and depression in this setting applied various coping strategies to meet their care needs. We observed that of the 19% employing a coping strategy, most (73%) reported borrowing from friends and relatives. Perhaps this choice can be explained by more favourable credit terms than they would receive from banks and other financial institutions, which require collateral and apply high interest rates. Participants borrowing from banks risk losing family assets if they are unable to make payments on time. Other HIV studies in India and Kenya found that PLWH sold household assets below market value and borrowed on high interest terms, risking long-term economic hardship for their families and households (Flores et al. 2008, Katana et al. 2020). Making HIV care free is an important step, but it is apparent from our results that people are continuing to incur costs of HIV and DD and often use coping mechanisms with further economic impact. Less detrimental strategies to spread costs, such as use of waivers and health insurance cover, need to be available options for individuals.

Our study fills an important gap by presenting the economic burden of DD and HIV for PLWH, but it does have some limitations. First, the results may be subject to recall bias by the respondents, despite limiting the recall period to 1 month. Second, the fact that the costs of HIV and DD were combined could make the results less useful for some purposes. This was a conscious choice because we did not believe it feasible to measure them separately without confusing participants, and because the main reason for the cost of illness analysis was to input it into a cost-effectiveness analysis. This would require any changes in HIV-related costs to be captured as well as DD, because the intervention could plausibly affect both. Third, about half of participants reported zero costs or missing data. According to our discussions with the field team, the missing data are best interpreted as zero costs in the vast majority of cases, in the context of how the questionnaire was delivered and recorded on paper. However, it is likely that our whole-sample estimates understate costs if some PLWH with missing values did incur costs but were not able to report them. This is why we also report the analyses in (Tables 3 and (5, to allow different users of our estimates to choose which is most appropriate for their purpose. Fourth, while working alongside a trial offers many advantages, it also means our sample is not representative of Uganda, and in particular is focused on rural areas. Finally, this was a facility-based study and those not accessing facilities were not represented.

The results may to some extent be generalizable to similar districts in Uganda, though part of our study took place during the COVID-19 pandemic. This may have resulted in underestimated costs due to restrictions on movement—another study in Uganda found that restrictions on public transport and high transport costs were barriers to accessing HIV services (Palattiyil et al. 2022). Furthemore, our estimates do not include those accessing services in the private sector, which has previously been shown to have higher cost than the public sector (Ekirapa et al. 2024), so our findings could be underestimated. Qualitative work on the economic burden of HIV and depression may help complement our findings by providing possible reasons for zero costs and poor utilization of facilities, as well as expanding on the choices of coping mechanisms. Broadening financial protection/coverage for HIV to also cover key comorbid diseases such as DD and tuberculosis could help to minimize OOP payments and mitigate worsening health outcomes. It would also be important to support local community services for DD so that these can be more accessible.

## Conclusion

The economic burden arising from care and management of HIV and depression is a significant public health concern. Although antiretrovirals are highly subsidized in most HIV clinics in Uganda, PLWH still incur considerable expenses relative to their incomes, due to comorbid conditions such as DD. This economic burden may potentially reinforce depressive symptoms on top of a vicious cycle of poverty. Social programmes to support individuals and families living with HIV are key to mitigating the economic and mental health impacts.

## Data Availability

Data from the HIV+D trial are available to bona fide researchers under the Medical Research Council and Uganda Virus Research Institute and London School of Hygiene & Tropical Medicine Uganda Research Unit Data Sharing Policy, which is available online at: https://apps.mrcuganda.org/mrcdatavisibility/Home/DataShare.

## Reflexivity statement

The authors include four women and seven men with professional experience spanning multiple levels. All authors specialize in various areas of public health and policy: seven in health economics, three in epidemiology and population health statistics, and one is a mental health expert. This study has benefitted greatly from the deep expertise of the authors in the United Kingdom, Uganda, and Kenya, as well as from their work across varied geographies within health economics and mental health.
